# Determining patient and primary care delay in the diagnosis of cancer – lessons from a pilot study of patients referred for suspected cancer

**DOI:** 10.1186/1471-2296-9-9

**Published:** 2008-01-30

**Authors:** Richard D Neal, Diana Pasterfield, Clare Wilkinson, Kerenza Hood, Matthew Makin, Helen Lawrence

**Affiliations:** 1 Cardiff University, North Wales Clinical School, UK; 2 Cardiff University, South East Wales Trials Unit, UK; 3 North East Wales NHS Trust, UK

## Abstract

**Background:**

There is no validated way of measuring diagnostic delay in cancer, especially covering patient and primary care delays. An instrument is needed in order to determine the effect of potential interventions to reduce delay and improve cancer morbidity and mortality.

**Methods:**

Development of a postal questionnaire tool to measure patient and primary care time responses to key symptoms and signs. The pilot questionnaire was sent to 184 patients with suspected cancer.

**Results:**

The response rate was only 85/184 (46.2%). Anxiety was cited as one reason for this low response. Patients returning questionnaires were more likely to be women and more likely to be younger. 84/85 (98.8%) provided consent to access medical records, and questions regarding health profile, smoking and socio-economic profile were answered adequately. Outcome data on their cancer diagnosis was linked satisfactorily and the question about GP-initiated investigations was answered well. Estimated dates for symptom duration were preferred for patient delays, but exact dates were preferred for primary care delays; however there was a significant amount of missing data.

**Conclusion:**

A more personal approach to the collection of data about the duration of symptoms in this group of people is needed other than a postal questionnaire. However elements of this piloted questionnaire are likely to figure strongly in future development and evaluation of this tool.

## Background

Mortality from cancer is worse in the UK than most other European countries [1]. Whilst there are several reasons for this, diagnostic delays and later stage at diagnosis are likely to be contributory factors. Interventions leading to reduced diagnostic delays and less advanced stage at diagnosis are therefore likely to lead to improved cancer survival figures and reduced morbidity. However delays at various points in the cancer diagnostic journey need mapping prior to the development and evaluation of such interventions. Diagnostic delays (perhaps better referred to as 'time to diagnosis' since there is not always a 'delay') may occur at any point in the cancer journey and can be divided into: pre-symptomatic delays, patient delays, primary care delays, referral delays, and secondary care delays [2,3]. The majority of cancer diagnoses are made in patients who present in primary care with symptoms [2,3], and the combination of patient and primary care delays are responsible for at least two thirds of total diagnostic delays; significantly more than referral and secondary care delays [2,3]. Recent UK government policy however, has focused primarily on minimising referral and secondary care delays [4-6], despite a lack of clear evidence for this approach [7-9].

There is no validated tool for determining cancer delays [10]. Studies reporting patient and primary care delays have often been methodologically poor and therefore inaccurate [10]. For example, they have used retrospective non-validated structured questionnaires (introducing the potential for recall bias), often with no reference to how retrospective they are [11] or have used case note review by surgeons who have used their own judgement to categorise delays [12]. Most studies that have attempted to report diagnostic delay have been poor at reporting the process of data collection.

The pilot study reported here is one step in the process of developing an appropriate tool to measure cancer delays from patients' perspectives. The aim of this pilot study was to achieve the following objectives:

1. To develop and pilot a short self-complete postal questionnaire for patients with symptoms suggestive of cancer, including a choice about the way the time to diagnosis question was asked

2. To develop and test a local protocol for patient recruitment

3. To determine the consent rate for access to medical records for this sample of patients

Once a tool is developed, appropriate interventions can be designed and trialled with the objective of reducing overall delays and reducing cancer mortality and morbidity.

## Methods

In this study, patient delay is defined as: 'the time between the onset of patients' experiences of symptoms which, in retrospect, they believe to have been due to the cancer, and presenting these symptoms to primary care', and primary care delay is defined as: 'the time between the presentation of these symptoms, or others that potentially may be cancer, in primary care and the onward referral of the patient to secondary care' [2].

### Identification of patients

We identified and recruited adult patients with suspected cancer from the North East Wales Trust. We worked closely with the Trust's Cancer Office to identify urgent suspected referrals. We set out to recruit 190 patients in order to ensure representation of sufficient numbers of differing cancer sites. Letters were sent in mid January 2005 to the Cancer Leads of the nine cancer sites and flyers forwarded to general practices that refer patients to North East Wales Trust informing them of the commencement of the research project. The Cancer Office identified, on a weekly basis, all adult patients, by cancer site, referred under the urgent suspected cancer guidance, over a prospective period for four weeks in early 2005. In total 184 patients were identified.

### Data collection from patients

Each identified patient was sent a pack which included a covering letter with a detailed information sheet inviting them to open the enclosed sealed envelope if they wished to take part in the research project or otherwise to return the pack to a Freepost address. The sealed envelope contained a covering letter welcoming the patient to the study, a consent form for access to their medical records and a questionnaire.

Part 1 of the questionnaire was specific to the patient's suspected cancer site. For each possible symptom for that cancer site, it asked the respondent how long before they saw their GP or practice nurse they had had their symptoms for, and how long elapsed between presenting their symptoms and referral. We deliberately asked for responses to these questions in *either *of two ways so that we could determine whether patients preferred to recall and enter an exact *or *an estimated date. The list of symptoms reflected the symptoms listed in the cancer referral guidelines for each specific cancer site [6]. Hence we knew in advance that this sample would be able to provide data about these symptoms.

Part 2 dealt with health profile (asking respondents whether they had certain conditions in the past two years), smoking habits, and socio-economic profile. General comments were invited.

Packs were sent as soon as possible after the urgent referral was received by the cancer office, and in most cases this would have been before they were seen in secondary care. Checks were made by the Cancer Office to ascertain whether patients had died before reminder letters were sent at three weeks and a second reminder with full study pack at six weeks. All documents other than the questionnaire were translated into Welsh.

### Cancer outcome data

Outcome data were obtained in July 2005 as either diagnosed or not diagnosed with cancer.

### Data analysis

The data analysis was predominantly descriptive, and analysed using SPSS; and is presented with the appropriate statistics.

## Results

### Response Rate

A total of 85 valid (questionnaires completed and analysable) (46.2%) responses were received. A further 56 were classed as declined responses (e.g. questionnaires returned unopened and messages via telephone stating that they did not wish to participate), and there was no response from 43 patients. The distribution between cancer sites is shown in Table 1. More women 66/133 (49.6%) had a valid response compared to 19/51 men (37.2%). Patients returning valid questionnaires were slightly younger (mean 58.9 years, median 56.9 years, with a range from 23.6 to 91.8 years and an inter-quartile range of 48.4 – 71.4 years) compared to those who did not (mean 62.8 years, median 66.7 years). Of the 85 valid responses, 49 (57.6%) responded to the first mailing, 24 (28.2%) to the first reminder, and 12 (14.1%) to the second reminder. Telephone calls were received from 16 patients, some with more than one comment. Nine of these comments were concerned with anxiety associated with receiving the questionnaire in some way; it was not possible to distinguish between the responses by patients with suspected compared with confirmed cancer because of the small numbers. The others concerned the provision of information about whether or not they were returning the questionnaire.

**Table 1:** Response by suspected cancer site

**Cancer site**	**Total Sent**	**Response (%)**	**Declined response**	**No response**
Breast	64	38 (59%)	6	20
Colorectal	29	10 (34%)	13	6
Gynaecological	22	11 (50%)	7	4
Head & Neck	15	7 (47%)	4	4
Lung	8	4 (50%)	3	1
Skin	16	7 (44%)	7	2
Upper GI	21	6 (29%)	11	4
Urological	9	2 (22%)	5	2

**Total**	184	85 (46%)	56	43

#### Consent to accessing medical records, Health profile, and Smoking & Socio-economic profile

Eighty two of the respondents (96.4%) provided consent to access their medical records. Non-response to the other questions was: Health Profile one respondent; smoking – five respondents; living with a spouse or partner – no respondents; ethnicity – two respondents; educational level – seven respondents; Employment status – two respondents. Detailed responses to these questions are available from the authors.

### Cancer diagnosis outcomes

Twenty nine of 184 (15.8%) patients were diagnosed with cancer. Thirteen (15.3%) of the responders were diagnosed and 16/99 non-responders (16.2%). By cancer site, there were 10 breast (16%), 5 colorectal (17%), 4 upper GI (19.0%), 3 urological (33.3%), 3 head & neck (20.0%), 2 gynaecological (9%), and 2 lung (25.0%).

### Symptom data

The numbers of symptoms reported on the questionnaires is shown in Table 2. This also shows how the respondents answered the questions about duration of symptoms. One suspected gynaecological cancer was identified through a screening trial and was asymptomatic. Because of the larger number of patients with breast cancer, and the corresponding number of symptoms we were able to break the response down further by symptom. This is shown in Table 3. The majority of recorded symptoms were those given in pre-determined categories. A small number of additional symptoms were recorded by individuals. This question was answered well, with only minimal missing data. For the question regarding duration of symptoms, exact answers were given for 25/152 (16.4%) symptoms, and estimated answers for 81/152 (53.3%); however for the question relating to when the symptoms were presented the figures were exact answers 45/152 (29.6%) and estimated answers 30/152 (19.7%).

**Table 2:** Response to exact and estimated time durations by cancer site

	Total number of symptoms reported	How long before your diagnosis did you notice this (symptom)?			How long before your diagnosis did you first tell your GP or nurse about this (symptom)?			
		
		Exact date	Estimated date	Data missing	Exact date	Estimated date	Data missing	n/a
Breast (n = 38)	52	9	30	13	21	11	13	7
Colorectal (n = 10)	16	2	10	4	7	5	3	1
Gynae (n = 11)	14	5	6	3	5	4	5	0
Head & Neck (n = 7)	16	0	9	7	1	2	11	2
Lung (n = 4)	13	3	2	8	0	2	11	0
Skin (n = 7)	17	1	8	8	3	2	11	1
Upper GI (n = 6)	21	5	13	3	8	3	5	5
Urology (n = 2)	3	0	3	0	0	1	2	0

Total (%)	152	25 (16.4%)	81 (53.3%)	46 (30.3%)	45 (29.6%)	30 (19.7%)	61 (40.1%)	16 (10.5%)

**Table 3:** Response to exact and estimated time durations for breast cancer by symptom

	How long before your diagnosis did you notice this (symptom)?			How long before your diagnosis did you first tell your GP or nurse about this (symptom)?			
	
	Exact date	Estimated date	Data missing	Exact date	Estimated date	Data missing	n/a
Lump (n = 30)	7	17	6	15	5	7	3
Pain (n = 19)	2	11	7	5	5	5	4
Others (n = 3)	0	2	1	1	1	1	0

Total	9	30	14	21	11	13	7

n = number of patients reporting this symptom

## Discussion

As a pilot study, the work reported here has achieved its three aims. Much has been gained from this work that will be taken forward to the next phase of the work.

The response to this survey was lower than we had initially hoped, and much lower than we would need for a future comprehensive study of delays. For example, *The National Survey of NHS Patients – Cancer *achieved a response of 74% [13], although this sampled patients with cancer rather than suspected cancer. Several factors may have contributed to our modest response. The questionnaire was overcomplicated, mainly because we chose to ask both exact and estimated dates as part of the piloting process, and this may have had an adverse effect on response. Some design features and wording on the pilot questionnaire were on reflection poor and again may have had an adverse effect, although it is impossible to quantify how much of a problem this may have caused. We suspect that the questionnaire may have generated anxiety in a small number of people; measures to reduce this may improve future response. This anxiety may have been generated in part by feelings of guilt at not presenting earlier that may have been precipitated by the tool, and may also have been generated by sampling patients with a 'suspected' diagnosis. There may have been a differential poor response due to age and ill-health related difficulties, however it was not possible to assess this due to lack of available data on non-responders.

We successfully demonstrated that the majority of this sample of people will consent to provide access to their medical records. This is similar to other work [14,15]. We were able to incorporate cancer outcome data. We also demonstrated that this sample of people will provide data regarding health, smoking, socio-economic profile, education, and GP-initiated investigations. However, a future version of the tool will use lessons learnt in this pilot to improve some of the category options. We identified wording in several places in the questionnaire that could be significantly improved in a future version.

In the analysis we had to make some assumptions about the data in order to calculate time periods from approximate dates. These included approximate date ranges that were coded as the longer time period (e.g. '3–4 weeks' was coded as 4 weeks); vague date ranges that were appropriately coded (e.g. 'a few months' was coded as 3 months); and approximate dates that were coded as the given figures (e.g. '14 days approx' was coded as 14 days). The other important issue was that 13/91 respondents provided no date of referral or provided an 'out of range' date; in these instances, the dates were entered from hospital records. In a very small number of instances, respondents answered both the exact date and the estimated date; when this occurred this was taken to be the exact date. If approximate dates are used in future versions of the tool, we will have to do similar in order to create continuous variables for analysis.

Most of the recorded symptoms were those expected from the urgent referral proformas, as expected. The use of free text to add additional symptoms worked well; none of the additional symptoms were prevalent enough to justify a category of its own in a future version.

As a general rule, estimated dates were preferred for patient delays, but exact dates were preferred for primary care delays. This may be because dates for primary care delays were both more recent and more memorable. However there was a significant amount of missing data; this may have been because the forms were confusing and will be improved in a future version. Whilst the numbers did not permit a detailed analysis of exact versus estimated by symptom (except for breast), it seemed to be the case that for more vague and undifferentiated symptoms (e.g. pain), estimated dates were preferred, whereas for more finite symptoms (e.g. breast lumps), exact dates were preferred. These findings will need to be taken into close consideration when designing a future version of the questionnaire.

## Conclusion

Our main conclusion from this study is that a different approach to the collection of data about the duration of symptoms in this group of people is needed other than a postal questionnaire. We are currently developing a researcher administered/facilitated version of the tool for administration within a health care setting, that we envisage will have a better response and create less anxiety. However the elements of the questionnaire pilot reported here are likely to figure strongly in future development and evaluation of a validated instrument for measuring patient and primary care cancer delays.

## Competing interests

The author(s) declare that they have no competing interests.

## Authors' contributions

The study was designed by all of the co-authors. HL identified patients eligible for the study. DP sent out questionnaires and reminders and entered the data. RDN analysed the data and wrote the first draft of the paper. All co-authors contributed to the draft of the paper.

## Pre-publication history

The pre-publication history for this paper can be accessed here:


